# The Herbal Compound Thymol Targets Multiple *Salmonella* Typhimurium Virulence Factors for Lon Protease Degradation

**DOI:** 10.3389/fphar.2021.674955

**Published:** 2021-08-26

**Authors:** Yong Zhang, Yan Liu, Jingjing Luo, Jing Jie, Xuming Deng, Lei Song

**Affiliations:** ^1^ Department of Respiratory Medicine, Key Laboratory of Organ Regeneration and Transplantation of the Ministry of Education, The First Hospital of Jilin University, Changchun, China; ^2^ Key Laboratory of Zoonosis, Ministry of Education, Institute of Zoonosis, College of Veterinary Medicine, Jilin University, Changchun, China

**Keywords:** type III secretion, *Salmonella*, anti-virulence, natural compounds, thymol

## Abstract

Many important bacterial pathogens are using the type III secretion system to deliver effectors into host cells. *Salmonella* Typhimurium (*S*. Typhimurium) is a pathogenic Gram-negative bacterium with the type III secretion system as its major virulence factor. Our previous studies demonstrated that thymol, a monoterpene phenol derivative of cymene, inhibited *S*. Typhimurium invasion into mammalian cells and protected mice from infection. However, the antibacterial mechanism of thymol is not clear. In this study, we revealed that thymol interferes with the abundance of about 100 bacterial proteins through proteomic analysis. Among the 42 proteins whose abundance was reduced, 11 were important virulence factors associated with T3SS-1. Further analyses with SipA revealed that thymol directly interacts with this protein to induce conformational changes, which makes it susceptible to the Lon protease. In agreement with this observation, thymol effectively blocks cell invasion by *S*. Typhimurium. Thus, thymol represents a class of anti-virulence compounds that function by targeting pathogenic factors for degradation.

## Introduction

Bacterial pathogens actively modulate host defense systems for successful colonization; such modulation is often achieved by virulence factors delivered into host cells by specialized protein secretion machineries belonging to systems such as the type III, type IV, and type VI secretion apparatuses (Costa et al., 2015). As mutants lacking such systems often are completely defective in virulence, these systems are valuable targets for the development of anti-infection agents (Rasko and Sperandio, 2010). Because inhibition of secretion systems involved in virulence does not impose a survival pressure on either the pathogen or the normal microflora, such anti-virulence compounds will less likely cause the development of resistance, and thus have been viewed as an ideal alternative to traditional antibiotics (Rasko and Sperandio, 2010).

*Salmonella enterica* serovar Typhimurium (*S*. Typhimurium) is an important foodborne pathogen. Recently, many antibiotic-resistant strains are constantly emerging worldwide. Therefore, it is urgent to develop new therapeutic methods to fight *Salmonella* infection. *S*. Typhimurium encoded two different machineries in T3SS, which is SPI-1 and SPI-2 (Agbor and McCormick, 2011). SPI-1 plays an important role in the process of bacterial invasion (Johnson et al., 2018). In the host cell, SPI-2 modulated the replication of bacteria (Johnson et al., 2018).

T3SS is a potent target for the development of therapeutic agents against microbes. Some compounds from natural products have been reported to target T3SS for the treatment of infection diseases (Pendergrass and May 2019; Lv et al., 2020; Sundin et al., 2020). Previous studies showed that thymol, a compound from traditional Chinese medicine (TCM), could reduce the biofilm formation (Cabarkapa et al., 2019) and disrupt the membrane integrity of *S*. Typhimurium *in vitro* (Chauhan and Kang, 2014). Studies based on proteomics and real-time PCR assay highlighted that thymol exposure significantly downregulated some main virulence genes of *S*. Typhimurium (Giovagnoni et al., 2020; Qi et al., 2020). Our previous study also confirmed the antibacterial effects of thymol in a mice model infected by *S*. Typhimurium (Zhang et al., 2018). However, the mechanism underlying the pharmacological activities of thymol is not clear. In this study, we reported that thymol strongly inhibited SipA delivered into HeLa cells but does not affect bacterial growth *via* using a reporter system of the activity of SPI-1. Further studies indicated that thymol functions by directly targeting multiple bacterial proteins, including important virulence factors for Lon protease degradation.

## Materials and Methods

### Ethic Statements

BALB/c mice were obtained from the Experimental Animal Center of Jilin University. All procedures involving animals were carried out in accordance with the Guidelines for the Use of Animals in Research, issued by the Jilin University.

### Chemicals

Unless otherwise noted, all chemicals used in this study were from Sigma. Among these, the purity of thymol used in all experiments was higher than 98.5% (Sigma, cat# T0501-500G). DMSO was used to dissolve thymol; the stock concentration is 100 mM.

### Bacterial Strains and Plasmid Construction

All strains of *S*. Typhimurium in this study were derived from the standard virulent strain SL1344 (Gulig and Curtiss, 1987). Bacteria were grown in the LB medium. Ampicillin (Amp, 100 μg/ml), kanamycin (Km, 50 μg/ml), or chloramphenicol (Cm, 30 μg/ml) was added into the medium for selection. The *lon* mutant derived from strain SL1344 (Boddicker and Jones, 2004) was a gift from Brady Jones (the University of Iowa), the strain harboring *hila::lacZ* fusion (Lin et al., 2008) was provided by James Slauch (the University of Illinois at Urbana-Champaign). The lactamase reporter plasmid was constructed by inserting the fragment of SipA–lactamase fusion into pQE30 between *BamHI* and *SalI* sites. The EspG–lactamase fusion for testing protein translocation by T3SS in enterohemorrhagic *E. coli* was similarly constructed. All plasmids used in this study are listed in Table 1. To make strains that express *SipA* and *SipB*, the open reading frame (ORF) of the genes were, respectively, inserted into the pKS3xFlag plasmid that carried both N-and C-terminal 3xFlag tags. This cassette was then inserted into the *pir* protein-dependent R6K vector pSR47S (Luo and Isberg, 2004), and the resulting plasmid was introduced into SL1344 by triparental mating with the *E. coli* helper strain HB101 (pRK600) (Luo and Isberg, 2004). Transconjugants were streaked onto the LB plate containing 15% sucrose. Strains in which the tag was inserted properly into the chromosome were identified by diagnostic PCR with the appropriate primer sets (Table 2).

**TABLE 1:** Strain or plasmids used in this study.

Strain or plasmid	Relevant genotypes	Sources
SL1344	Wild-type serovar Typhimurium	Liu et al. (2015); Zhou et al. (1999)
JS749	*att::*pDX1*::hilA-lacZ*	
JS751	*att::pDX1::sopB-lacZ*	
JS752	*att::pDX1::sicA-lacZ*	
BJ3410	*Δlon*	
	*ΔinvA*	Zhou et al. (1999)
DL1101	*ΔclpP*	This study
DL1102	*ΔsipA*	This study
DL1103	sipA*3flag	This study
DL1104	sipB*3flag	This study
DL1105	*WT::sipA-pzlq*	This study
DL1106	*WT::sipA(1-270)-pzlq*	This study
DL1107	*WT::sipA-(27-270)-pzlq*	This study
DL1108	*WT::sipA-(497-669)-pzlq*	This study
DL1109	*WT::sipB-pzlq*	This study
DL1110	*WT::sipA-6p-1*	This study
DL1111	*WT::hilC-6p-1*	This study
DL1112	*WT::hilD-6p-1*	This study
DL1113	*WT::prgK-6p-1*	This study
DL1114	*WT::prgH-6p-1*	This study
DL1115	*WT::sipA-lactamase*	This study
Plasmids		
pSR47s	*ori*R6K, *ori*T RP4, Kan^R^, *SacB*	Luo and Isberg (2004)
PKS	Amp, construct used for in-frame knock in 3*flag	This study
pRK600	Cm^r^ ColEloriV RP4oriT, helper plasmid in triparental mating	Invitrogen
PZLQ	Expression vector with the *tac* promoter	Luo and Farrand (1999)
pKD3	Construct used for in-frame deletion of *sipA* and *sipB*	Invitrogen
pCP20	bla cat cI857 PRflppSC101 *ori*TS	Invitrogen
pKD46	blaPBADgam bet exopSC101 *ori*TS	Invitrogen
pET-32a	For expression His_6_-tagged protein *S.* Typhimurium	Invitrogen
pGEX-6p-1	For expression GST-tagged protein *S.* Typhimurium	Invitrogen
pDL1201	pZLQ::*sipA*	This study
pDL1202	pZLQ::*sipB*	This study
pDL1203	pZLQ::*sipA(1-270)*	This study
pDL1204	pZLQ::*sipA(27-270)*	This study
pDL1205	pZLQ::*sipA(497-669)*	This study
pDL1206	pGEX-6p-1::*sipA*	This study
pDL1207	pGEX-6p-1:: *sipA(1-270)*	This study
pDL1208	pGEX-6p-1:: *sipA(27-270)*	This study
pDL1209	pGEX-6p-1:: *sipA(497-669)*	This study
pDL1210	pGEX-6p-1:: *prgK*	This study
pDL1211	pGEX-6p-1:: *prgH*	This study
pDL1212	pGEX-6p-1:: *hilC*	This study
pDL1213	pGEX-6p-1:: *hilD*	This study
pDL1214	pEX233::*sipA*	This study
pDL1215	pET32a::*lon*	This study

**TABLE 2:** Primers used in this study.

Primer name	Sequence (restriction enzyme sites are underlined)	Note
SL1101	GTCGAG​CTCGTG​GTC​ACG​TCA​GAA​AAG​GGC​A	sipA up Sac I
		Knock-in 3*flag
SL1102	TCCCCC​GGGACG​CTG​CAT​GTG​CAA​GCC​ATC​AAC​G	sipA up Sma I
		Knock-in 3*flag
SL1103	CGCCTC​GAGTAA​TTA​ACC​GGG​AAA​GAT​GCG​ATG​A	sipA down Sac I Knock-in 3*flag
SL1104	CATGGT​ACCGTT​CAC​CAT​TAA​TCA​CCA​TAA	sipA down Sma I Knock-in 3*flag
SL1105	CTTGTC​GACGTT​CAC​CAT​TAA​TCA​CCA​TAA	sipA down Sal I Knock-in 3*flag
SL1106	GTCGAG​CTCACC​ATT​GTC​AGC​GTT​GTG​GC	sipB up Sac I
		Knock-in 3*flag
SL1107	TCCCCC​GGGTGC​GCG​ACT​CTG​GCG​CAG​AAT​AAA​A	sipB up Sma I
		Knock-in 3*flag
SL1108	CGCCTC​GAGTAA​AAA​CTG​CCA​AAA​TAA​AGG​GAG​A	sipB down Xho I Knock-in 3*flag
SL1109	CATGGT​ACCCTT​CAT​ATT​TAA​CGA​TTT​CAG​AGA​A	sipB down Kpn I Knock-in 3*flag
SL1110	CTTGTC​GACCTT​CAT​ATT​TAA​CGA​TTT​CAG​AGA​A	sipB down Sal I Knock-in 3*flag
SL1111	CGCGGA​TCCGTT​ACA​AGT​GTA​AGG​ACT​CAG​C	sipA 5^'^ BamH I
SL1112	CTTGTC​GACTTA​ACG​CTG​CAT​GTG​CAA​GCC​ATC​A	sipA 3^'^ Sal I
SL1113	CGCGGA​TCCGTA​AAT​GAC​GCA​AGT​AGC​ATT​A	sipB 5^'^ BamH I
SL1114	CTTGTC​GACTTA​TGC​GCG​ACT​CTG​GCG​CAG	sipB 3^'^ Sal I
SL1115	CGCGGA​TCCGTT​ACA​AGT​GTA​AGG​ACT​CAG​CC	sipA (1–270) 5^'^
		BamH I
SL1116	CGCGGA​TCCCTT​GCG​GCG​AAT​CTT​TCC	sipA (27–270) 5^'^ BamH I
SL1117	CTTGTC​GACTTT​AGG​GTC​AGG​CAG​TTT​ATC​T	sipA (1–270) 3^'^ Sal I
SL1118	CGCGGA​TCCAAT​AAA​GCC​GGT​ACG​ACC​GAT	sipA (497–669) 5^'^
		BamH I
SL1119	CTTGTC​GACAGG​TTT​ATT​CAG​GTA​TGA​CTC​GTA​AG	sipA (497–669) 3^'^Sal I
SL1120	CGCGGA​TCCGTT​ACA​AGT​GTA​AGG​ACT​CAG​CC	sipA 5^'^ BamH I
SL1121	CCGCTC​GAGTTA​ACG​CTG​CAT​GTG​CAA​GCC	sipA 3^'^ Xho I
SL1122	CGCGGA​TCCATT​CGT​CGA​TAT​CTA​TAT​ACT​TTT​CTG​C	prgK 5^'^ BamH I
SL1123	CCGGAA​TTCCTA​TTC​ATT​TGA​CGA​TTT​CGC​CTT​A	prgK 3^'^ EcoR I
SL1124	CGCGGA​TCCGCG​ATG​TCG​ATT​GCA​ACT​ATT​GTC​CCT​GA	prgH 5^'^ BamH I
SL1125	CCGGAA​TTCCGG​TCA​TGA​GCG​TAA​TAG​CGT​TTC​AAC​AG	prgH 3^'^ EcoR I
SL1126	CGCGGA​TCCGTA​TTG​CCT​TCA​ATG​AAT​AAA​TCA	hilC 5^'^ BamH I
SL1127	CCGGAA​TTCTCA​ATG​GTT​CAT​TGT​ACG​CAT​AAA	hilC 3^'^ EcoR I
SL1128	CGCGGA​TCCGAA​AAT​GTA​ACC​TTT​GTA​AGT​AAT​AGT​CA	hilD 5^'^ BamH I
SL1129	CCGGAA​TTCTTA​ATG​GTT​CGC​CAT​TTT​TAT​GAA​TG	hilD 3^'^ EcoR I
SL1130	CGCGGA​TCCCTT​GCG​GCG​AAT​CTT​TCC	sipA (27–270)
		5^'^BamH I
SL1127	CCG​GAA​TTC​TCA​ATG​GTT​CAT​TGT​ACG​CAT​AAA	hilC 3^'^ EcoR I
SL1128	CGC​GGA​TCC​GAA​AAT​GTA​ACC​TTT​GTA​AGT​AAT​AGT​CA	hilD 5^'^ BamH I
SL1129	CCG​GAA​TTC​TTA​ATG​GTT​CGC​CAT​TTT​TAT​GAA​TG	hilD 3^'^ EcoR I
SL1130	CGC​GGA​TCC​CTT​GCG​GCG​AAT​CTT​TCC	sipA (27–270)
		5^'^BamH I
SL1131	CTTCTC​GAGTTT​AGG​GTC​AGG​CAG​TTT​ATC​T	sipA (27–270)
		3^'^Xho I
SL1132	CGCGGA​TCCAAT​AAA​GCC​GGT​ACG​ACC​GAT	sipA (497–669)
		5^'^ BamH I
SL1133	CTTCTC​GAGAGG​TTT​ATT​CAG​GTA​TGA​CTC​GTA​AG	sipA (497–669)
		3^'^ Xho I
SL1134	CCGGAA​TTCAAT​CCT​GAG​CGT​TCT​GAA​CGC	Lon 5^'^ EcoR I
SL1135	CCGCTC​GAGCTA​TTT​TGC​GGT​TAC​AAC​CTG​C	Lon 3^’^ Xho I
SL1136	ATG​GTT​ACA​AGT​GTA​AGG​ACT​CAG​CCC​CCC​GTC​ATA​A	sipA 5^'^Knock-out
	TGC​CAG​GTA​TGC​AGA​CGA​TTG​TGT​AGG​CTG​GAG​CTG​C	
SL1137	TTA​ACG​CTG​CAT​GTG​CAA​GCC​ATC​AAC​GGT​AGT​AAT​A	sipA 3^’^ Knock-out
	ACC​CGA​TCC​ACT​TAA​CGG​CTG​ACA​TGG​GAA	
SL1138	ATG​TCA​TAC​AGC​GGA​GAA​CGA​GAT​AAT​TTG​GCC​CCT​C	clpP 5^'^ Knock-out
	ATATGGCGCTGGTGCC	
SL1139	TCA​ATT​ACG​ATG​GGT​CAA​AAT​TGA​GTC​AAC​CAA​ACC​G	clpP 3^'^ Knock-out
	TAC​TCT​ACC​GCA​CTT​AAC​GGC​TGA​CAT​GGG​AA	

The deletion of *sipA* and *clpP* was performed with the λ Red and FLP-mediated site-specific recombination (Datsenko and Wanner, 2000). Primers containing 45-base corresponding to each end of the gene of interest linked to a 20-base of the FRT flanked the antibiotic resistance cassette on pKD3 were used to amplify the fragment containing chloramphenicol. A 100-ml culture was established from an overnight culture of SL1344 carrying pKD46 to an OD_600_ of 0.1. L-arabinose was added at a final concentration of 10 mM. The culture was further incubated at 30°C till OD_600_ reached 0.4; cells were then collected to prepare electrocompetent cells by washing twice with ice cold PBS, followed by a wash with 10% ice-cold glycerol. Electroporation was performed using 50 μl cells and 1 μg of the PCR product. After recovery at 37°C in 1 ml SOC for 2 h in a shaker (200 rpm), the cells were plated onto the LB plate containing chloramphenicol. The mutants were identified by diagnostic PCR with the appropriate primers (Table 2). The chloramphenicol resistance cassette was eliminated by introducing pCP20, a temperature-sensitive plasmid that expresses the FLP recombinase (Cherepanov and Wackernagel, 1995).

### Experimental Design and Statistical Rationale for Proteomic Analysis

Bacterial cells from 1 ml culture grown in the LB medium containing 300 mM NaCl to OD_600_ of 0.9 were used. Thymol was added to identical cultures at the concentration of 0.2 mM. Total bacterial lysates were run on a 10% SDS-PAGE and stopped once the bromophenol blue dye reached 1 cm below the stacking gel. The whole gel was equally cut into five horizontal slices and subjected to in-gel protein digestion with trypsin as described previously (Hu et al., 2014). Briefly, samples of digested peptides were dissolved in HPLC-grade water and were analyzed on a nanoflow reversed-phase liquid chromatography (EASY-nLC1000, Thermo Scientific) coupled with a linear ion trap mass spectrometer (LTQ Velos Pro, Thermo Scientific) in a data-dependent mode. The 75 μm × 150 mm capillary column was packed with 5 μm, 100 Å Magic C18AQ silica-based particles (Michrom BioResources Inc., Auburn, CA). The column had an integrated electrospray tip that was made in-house with a laser-based puller (Model P-2000, Sutter Instruments). The LC separation was carried out with the following gradient: solvent B (100% acetonitrile and 0.1% formic acid) was started at 7% for 3 min, and then raised to 35% in 40 min; subsequently, solvent B was rapidly increased to 90% in 2 min and maintained for 10 min before 100% solvent A (97% H_2_O, 3% acetonitrile, and 0.1% formic acid) was used for column equilibration. One full MS scan (*m/z* 350–1,500) was followed by MS/MS analyses of the top 10 most intense ions. Dynamic exclusion was enabled with a repeat duration of 12 s and exclusion duration of 6 s. With three pairs of biological replicates (control and thymol-treated samples) and five gel fractions per sample, we carried out 30 (3 × 2×5) LC-MS/MS experiments.

### Proteomic Data Processing

LC-MS/MS raw files were searched against *S*. Typhimurium LT2 protein database (strain LT2/SGSC1412/ATCC 700720, July 11, 2011, 5,199 sequences) downloaded from UniProt (http://www.uniprot.org/) with Mascot (version 2.3.02, Matrix Science Inc.). The following settings were used for database search: 1.5 Da precursor mass error tolerance and 0.8 Da fragment mass error tolerance. A maximum of two missed cleavage sites were allowed. Oxidation (M) was set as a variable modification. The peptide and protein identifications were filtered to achieve a false discovery rate (FDR) < 1%. Protein relative abundance between different samples was assessed using spectral counting (Liu et al., 2004), which represents the total number of repeated peptide identifications for a given protein and provides a semiquantitative assessment of protein abundance.

A protein fold change between paired samples was calculated by dividing the average spectral counts of triplicate measurements. Positive or negative values indicate higher or lower levels in the thymol-treated samples compared to untreated cells. Differences with average fold changes ≥1.5 or ≤0.67 and *p* value ≤ 0.05 were considered significant. Paired Student’s *t*-test was performed on data from three biological replicates.

### Immunoblotting

The HilA-specific antibody (Sang et al., 2016) is a generous gift from Yufeng Yao (Shanghai Jiao Tong University). Mouse anti-SipA antibodies were produced in our animal facility. Briefly, 1 mg of His_6_-SipA was emulsified with equal volume of complete Freund’s adjuvant and was injected to five mice intracutaneously 4 times a month at 7 day intervals. The presence of SipA-specific antibodies in the sera of the immunized mice was tested, and those that were reactive were used for affinity IgG purification. To test protein stability, overnight cultures of wild-type *S*. Typhimurium and the relevant mutants expressing the indicated recombinant or tagged proteins were diluted at 1:20 in 2 ml LB and grown for 4 h at 37°C in a shaker (200 rpm); thymol was added to identical cultures at indicated concentrations. OD_600_ values were used to estimate the number of bacterial cells collected for subsequent experiments. Cells pelleted by centrifugation at 12,000 g for 20 min were resuspended with the 100 μl 1x SDS sample buffer. The samples were boiled for 5 min prior to SDS-PAGE. Proteins transferred onto nitrocellulose membranes were incubated in 5% BSA supplemented with the appropriate antibodies, including anti-SipA antibody (1:500), anti-HilA (1:1,000), anti–β-lactamase (Abcam) (1:170), and antibody for metabolic enzyme isocitrate dehydrogenase (ICDH, 1:20,000) (Xu et al., 2010). In all cases, the signals were detected with a secondary antibody conjugated to horseradish peroxidase by the enhanced chemiluminescence (ECL) method.

### *In Vitro* Protease Degradation Assay

To analyze the degradation of SipA and its derivatives in reactions with defined components, 400 μg His_6_-Lon SipA or equivalent volumes of buffer were added into reactions containing SipA or its derivatives purified from cells in cultures with or without thymol. The reaction was initiated by 1 mM ATP followed by incubation in 37°C for 30 min. Identical reactions terminated by SDS sample buffers immediately after adding ATP were used as input controls. Proteins of interest in the reactions were detected by Coomassie bright blue staining or by immunoblotting. For cell-free assays, total protein lysates of *S*. Typhimurium were prepared from the 100 ml cultures grown at the exponential phase (OD_600_ = 0.8) by sonication followed by centrifugation at 10,000 g for 15 min. 400 μg purified GST-SipA expressed in cultures with or without thymol was added to the lysates. After incubation for the indicated time duration, the protein was detected by immunoblotting with specific antibodies.

### β-galactosidase Assay

β-galactosidase activity was determined as described by Luo et al. before (Luo and Farrand, 1999). Briefly, JS749 strain was cultured overnight (Lin et al., 2008) and diluted 1:20 in LB broth with or without thymol at the indicated concentrations. Bacteria density was determined by OD_600_ measurement, and the bacteria were harvested by centrifugation (12,000 *g,* 10 min). Cells resuspended in Z-buffer were mixed with 20 μl 0.1% freshly configured SDS solution and 40 μl chloroform. 100 μl cells lysed on a vortex for 10 s were transferred to a 96-well plate, and 20 μl of ONPG (4 mg/ml) in Z-buffer was then added into the lysates to initiate the reaction. The mixtures were incubated at room temperature for 10 min before the reactions were terminated with 50 μl of 1 M Na_2_CO_3_. A_420_ values were measured by a plate reader.

### The Binding Between SipA and Thymol

5 ml cultures of BL21 (DE3) derivatives harboring the plasmids directing the expression of GST-SipA_27-270_ or GST-SipA_497-669_ were induced with IPTG. Alkyne thymol was added to identical cultures, and the cultures were incubated at 20°C for 16 h. The bacterial cells were suspended with 1 ml 0.1% SDS solution containing 50 mM triethanolamine (TEA, Sigma), 150 mM NaCl, 1xEDTA-free protease inhibitor cocktail (Roche), 5 mM PMSF (Sigma), and 0.1 μL benzonase (Sigma). Cells were lysed by sonication for 3 min, and the lysates were then incubated on ice for 30 min. After removing the debris by centrifugation at 12,000 *g* for 10 min, the supernatant was loaded onto a glutathione-Sepharose column for protein purification. The proteins of interest were eluted with the elution buffer (50 mM Tris-Cl pH 8.0, 0.4 M NaCl, 25 mM reduced glutathione, and 0.1% Trition X-100, 1 mM DTT). 400 μg of protein in the elution buffer was used to detect the interactions. 12% SDS buffer (12% w/v in 50 mM TEA and 150 mM NaCl) was added to the protein solution to bring the final SDS concentration to 4% in a total volume of 44.5 μl. Click chemistry reaction was performed as described (Speers et al., 2003; Tsou et al., 2016). Briefly, a master mixture of click chemistry reagents that contains 1 μl 5 mM Azide-fluor 488 (Sigma), 1 μl 50 mM TCEP (Tris(2-carboxyethyl) phosphine), 2.5 μl 2 mM TBTA Tris(benzyltriazolylmethyl) amine, and 50 mM CuSO_4_ was prepared. 5.5 μl of the master mixture was added to each protein sample for a final volume of 50 μl. After vigorous mixing on a votex, the reactions were incubated at room temperature for 1 h. Finally, unreacted molecules were removed by chloroform/methanol extraction, and the samples were resolved by SDS-PAGE. Proteins were detected by Coomassie bright blue staining or immunoblotting. Azido-rhodamine bound to alkyne-thymol was detected by fluorescence scanning with a Typhoon 7,000 imager (Amersham Biosciences).

### Circular Dichroism Spectroscopy

GST-SipA_27-270_ and GST-SipA_497-669_ purified from cells or lysates with or without thymol were analyzed by circular dichroism spectroscopy for structural changes. CD spectroscopy analysis of GST-SipA_27-270_ and GST-SipA_497-669_ was performed with protein concentrations of 7.9 and 7.3 μM, respectively, in phosphate-buffered saline (135 mM NaCl, 2.7 mM KCl, 1.5 mM KH_2_PO_4_, and 8 mM Na_2_HPO_4_). The spectrum was obtained at 25°C by averaging over five scans with a step size of 1 nm and an averaging time of 2.5 s on a Chirascan-plus CD spectrometer. Measurements were performed in a Hellma quartz cuvette of path length 0.1 cm. Helix content was calculated from the molar ellipticity at 208 nm.

## Results

### Thymol Treatment Alters the Level of About One Hundred Proteins in *S*. Typhimurium

To determine the mechanism underlying the inhibition of T3SS-1 by thymol, we performed proteomic analyses of *S*. Typhimurium cells by liquid chromatography–tandem mass spectrometry (LC)-MS/MS. A total of 2,875 proteins were detected in all three independent experiments in which at least two tryptic peptides were scored for each protein (Lau et al., 2014). Consistent with the observation that thymol used at less than 0.4 mM did not detectably affect bacterial viability, the level of most of the identified proteins (2,764 or >96%) remained unchanged in cells grown in the presence of 0.2 mM thymol. The abundance of 65 proteins, including many predicted to be involved in metabolism, was significantly increased upon thymol treatment. For example, TorA and TorC, two enzymes involved in trimethylamine-N-oxide metabolism, exhibited the highest induction, at 72- and 13-fold, respectively. It is possible that thymol triggers the expression of some regulatory proteins, but the mechanism underlying such induction of these proteins needs further investigation.

We also observed significant reduction of 46 proteins in cells grown in 0.2 mM thymol (Table 3). Importantly, 11 of these 46 proteins were virulence factors associated with the SPI-1, including its effectors SipA, SipB, SipD, and SopB whose reduction was more than 7-fold (Table 4). The level of HilA and HilD, regulatory proteins that directly control the expression of the invasion genes, including many effectors (Bajaj et al., 1995; Thijs et al., 2007), was reduced by about 9- and 3.7-fold, respectively, suggesting that the reduction associated with the effectors can be caused by lower transcription or lower protein stability, or a combination of both.

**TABLE 3:** Protein with decreased abundance in thymol-treated samples.

		Abundance^a^			
Gene	Protein description	Untreated	Thymol-treated	Fold change^b^	*p*-value^c^
sipA	Cell invasion protein sipA OS = *Salmonella* Typhimurium GN = sipA PE = 1 SV = 2	95	4	16.4	0.001
sipB	Cell invasion protein sipB OS = *Salmonella* Typhimurium GN = sipB PE = 1 SV = 1	123	5	12.6	0.003
sipD	Cell invasion protein sipD OS = *Salmonella* Typhimurium GN = sipD PE = 2 SV = 1	37	2.5	9.1	0.002
hilA	Transcriptional regulator hilA OS = *Salmonella* Typhimurium GN = hilA PE = 2 SV = 2	29	3	8.7	0.001
tdcB	Threonine dehydratase catabolic OS = *Salmonella* Typhimurium GN = tdcB PE = 1	45	5.5	8.7	0.002
sicA	Chaperone protein sicA OS = *Salmonella* Typhimurium GN = sicA PE = 1 SV = 1	20	2	8.6	0.002
tdcC	Threonine/serine transporter tdcC OS = *Salmonella* Typhimurium GN = tdcC PE = 3	28	3.5	7.7	0.003
sopB	Inositol phosphate phosphatase sopB OS = *Salmonella* Typhimurium GN = sopB PE = 1SV = 2	93	3	7.1	0.022
sptP	Effector protein sptP OS = *Salmonella* Typhimurium GN = sptP PE = 1 SV = 1	22	2.5	6.5	0.005
tdcG	L-serine deaminase OS = *Salmonella* Typhimurium GN = tdcG PE = 4 SV = 1	18	3.5	5.2	0.003
STM4498	Putative inner membrane protein OS = *Salmonella* Typhimurium GN = STM4498 PE = 4SV = 1	12	2.5	4.8	0.003
tdcD	Propionate kinase OS = *Salmonella* Typhimurium GN = tdcD PE = 1 SV = 2	9	2	4.5	0.001
motB	Chemotaxis protein motB OS = *Salmonella* Typhimurium GN = motB PE = 3 SV = 1	14	2	4.5	0.030
prgH	Protein prgH OS = *Salmonella* Typhimurium GN = prgH PE = 4 SV = 1	34	6	4.2	0.029
hsdR	Endonuclease R OS = *Salmonella* Typhimurium GN = hsdR PE = 4 SV = 1	17	6	3.8	0.017
invG	Protein invG OS = *Salmonella* Typhimurium GN = invG PE = 3 SV = 3	108	26	3.8	0.018
hilD	Transcriptional regulator hilD OS = *Salmonella* Typhimurium GN = hilD PE = 4 SV = 2	12	1.5	3.7	0.030
melA	Alpha-galactosidase OS = *Salmonella* Typhimurium GN = melA PE = 3 SV = 2	9	2.5	3.6	0.001
ydiJ	Putative oxidase OS = *Salmonella* Typhimurium GN = ydiJ PE = 4 SV = 1	15	4.5	3.3	0.005
Rob	Transcriptional regulator OS = *Salmonella* Typhimurium GN = rob PE = 4 SV = 1	24	7	3.3	0.008
pduJ	Propanediol utilization protein OS = *Salmonella t* Typhimurium GN = pduJ PE = 4 SV = 1	7	2	3.3	0.020
yiaO	Putative dicarboxylate-binding periplasmic protein OS = *Salmonella* Typhimurium GN = yiaO PE = 4 SV = 1	5	1.5	3.0	0.015
STM2504	Putative cytoplasmic protein OS = *Salmonella* Typhimurium GN = STM2504 PE = 4	6	2	3.0	0.015
mreC	Rod shape-determining protein OS = *Salmonella* Typhimurium GN = mreC PE = 4 SV = 1	5	1.5	3.0	0.015
prgI	Protein prgI OS = *Salmonella* Typhimurium GN = prgI PE = 1 SV = 1	7	2	3.0	0.020
glnE	Glutamate-ammonia-ligase adenylyltransferase OS = *Salmonella* Typhimurium GN = glnE PE = 3 SV = 1	10	4	3.0	0.038
STM2529	Putative anaerobic dimethylsulfoxide reductase OS = *Salmonella* Typhimurium GN = STM2529 PE = 4 SV = 1	7	2.5	2.8	0.013
cheR	Chemotaxis protein methyltransferase OS = *Salmonella* Typhimurium GN = cheR PE = 1SV = 1	7	2.5	2.8	0.013
sixA	Phosphohistidine phosphatase OS = *Salmonella* Typhimurium GN = sixA PE = 4 SV =	7	2.5	2.8	0.013
Cfa	Cyclopropane fatty acyl phospholipid synthase OS = *Salmonella* Typhimurium GN = cfa PE = 4 SV = 1	8	2.5	2.7	0.038
tnpA	TnpA OS = *Salmonella* Typhimurium GN = tnpA PE = 4 SV = 1	5	1.5	2.6	0.032
yjiY	Putative carbon starvation protein OS = *Salmonella* Typhimurium GN = yjiY PE = 4	13	5	2.5	0.015
citE	Citrate lyase beta chain (Acyl lyase subunit) OS = *Salmonella* Typhimurium GN = citEPE = 4 SV = 1	15	6	2.4	0.007
degS	Periplasmic serine endoprotease OS = *Salmonella* Typhimurium GN = degS PE = 4	12	5	2.4	0.007
Bla	Beta-lactamase SHV-2 OS = *Salmonella* Typhimurium GN = bla PE = 3 SV	5	2	2.4	0.020
exoX	DNA exonuclease X OS = *Salmonella* Typhimurium GN = exoX PE = 4 SV = 1	4	1.5	2.4	0.020
hybF	Probable hydrogenase nickel incorporation protein hybF OS = *Salmonella* Typhimurium GN = hybF PE = 3 SV = 1	4	1.5	2.4	0.020
potE	APC family, putrescine/ornithine antiporter OS = *Salmonella* Typhimurium GN = potEPE = 4 SV = 1	6	2	2.4	0.020
waaK	Lipopolysaccharide 1,2-N-acetylglucosaminetransferase OS = *Salmonella* Typhimurium GN = waaK PE = 3 SV = 1	8	3.5	2.4	0.020
yhjE	Putative MFS family transport protein OS = *Salmonella* Typhimurium GN = yhjE PE = 4SV = 1	4	1.5	2.4	0.020
yrbK	Putative inner membrane protein OS = *Salmonella* Typhimurium GN = yrbK PE = 4	5	2	2.4	0.020
rfbI	Protein rfbI OS = *Salmonella* Typhimurium GN = rfbI PE = 4 SV = 1	29	11	2.3	0.035
srfB	SrfB (SsrAB activated gene) OS = *Salmonella* Typhimurium GN = srfB PE = 4 SV = 1	63	27	2.2	0.017
argH	Argininosuccinate lyase OS = *Salmonella* Typhimurium GN = argH PE = 3 SV = 1	9	4	2.1	0.004
ygjR	Putative dehydrogenase OS = *Salmonella* Typhimurium GN = ygjR PE = 4 SV = 1	10	5.5	2.1	0.049
ybiB	Putative transferase OS = *Salmonella* Typhimurium GN = ybiB PE = 4 SV = 1	10	4.5	2.1	0.002

a Averaged spectral counts from three biological replicates.

b Fold change in protein abundance; positive or negative values indicate higher or lower levels in the thymol-treated samples comparing to untreated cells.

c p-values were calculated using paired Student’s *t*-test.

**TABLE 4:** Thymol affects the abundance of *Salmonella* virulence factors.

Gene	Protein description	Abundance^a^		Fold change^b^	*p*-value^c^
		Untreated	Thymol-treated		
sipA	Effector protein	95	4	16.4	0.001
sipB	Effector protein	123	5	12.6	0.003
sipD	Effector protein	37	2.3	9.1	0.002
hilA	Transcriptional regulator HilA	29	3	8.7	0.001
sicA	Chaperone protein SicA	20	2	8.6	0.002
sopB	Effector protein	93	3	7.1	0.022
sptP	Effector protein	22	2.5	6.5	0.005
prgH	Needle complex proteins PrgH	34	6	4.2	0.029
invG	Invasion protein	108	26	3.8	0.017
hilD	Transcriptional regulator HilA	12	1.5	3.7	0.030
prgI	Needle complex proteins PrgI	7	2	3	0.020

a Averaged spectral counts from three biological replicates.

b Fold change in protein abundance: the average of fold change of three independent experiments.

c *p*-values were calculated using paired Student’s *t*-test.

### Thymol Reduced the Cellular Levels of Several Proteins Associated With T3SS-1

**FIGURE 1:**
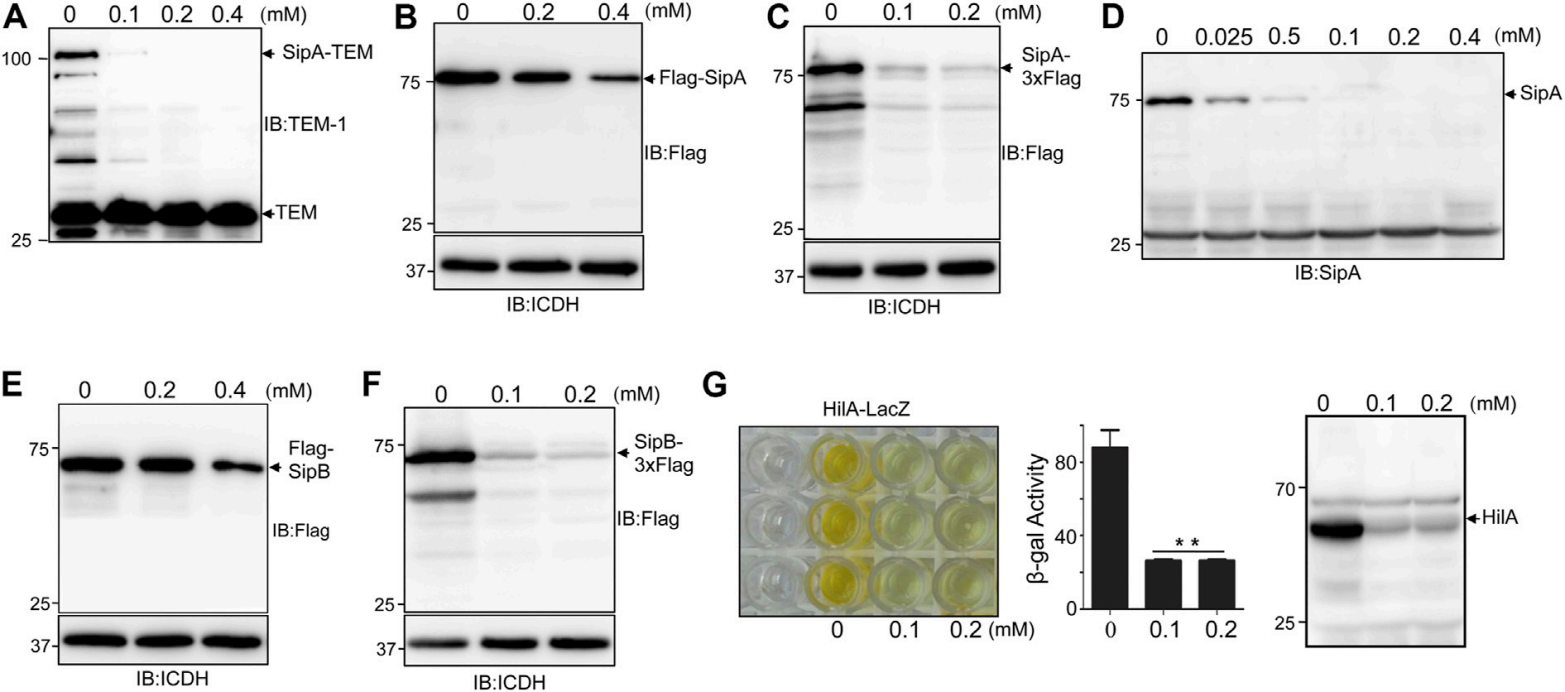
Treatment by thymol reduced the protein level of several virulence factors associated with SPI-1. **(A–D)** Thymol affects the cellular level of SipA or its fusion to various tags expressed by different promoters. Cultures of *S. typhimurium* harboring the construct that directs the expression of SipA–lactamase **(A)**, Flag–SipA from a high-copy plasmid driven by the *tac* promoter (Luo and Farrand, 1999) **(B)**, SipA–3xFlag from the chromosome **(C)**, or its endogenous form was treated with the indicated concentrations of thymol for 4 h. Cleared cell lysates resolved by SDS-PAGE were probed for the fusion or SipA by antibodies specific for the tag or the protein itself. Irrelevant proteins recognized by the antibody **(A,D)** and the ICDH were probed and served as loading controls **(B,C)**. **(E–F)** The induction of SipB degradation by thymol. Cultures of *S.* Typhimurium harboring a high-copy plasmid expressing Flag–SipB **(A)** or SipB–3xFlag on the chromosome were incubated with the indicated concentrations of thymol for 4 h prior to cell lysis and immunoblotting with the Flag-specific antibody. The metabolic enzyme ICDH was probed as loading control. **(G)** Thymol affects the stability of HilA. Cultures of an *S*. Typhimurium strain expressing a HilA–LacZ fusion from the chromosome prepared by including thymol at the indicated concentrations were measured for β-galactosidase activity. The reduction in the yellow color product generated by β-galactosidase–induced ONPG hydrolysis (left) or the enzymatic activity in miller unit (middle panel) was shown. Lysates of wild-type bacteria treated with thymol were probed by immunoblotting with a HilA-specific antibody (right panel). The experiments were repeated at least three times each with independent biological samples; bar, s.e.m. (*n* = 3); **, *p* < 0.01.

To distinguish whether thymol directly affects the stability of these SPI-1 effectors, we focused on SipA by first examining levels of the SipA–lactamase fusion expressed from the synthetic *tac* promoter (Luo and Farrand, 1999). Whereas untreated cells expressed readily detectable fusion protein; inclusion of 0.1 mM thymol in the bacterial cultures drastically reduced the protein level of the fusion and 0.2 mM rendered the protein completely undetectable; thymol at concentrations as high as 0.4 mM did not detectably affect the level of the β-lactamase expressed from the plasmid backbone (Figure 1A), suggesting that thymol targets SipA but not β-lactamase. Similar results were obtained when a Flag–SipA fusion was expressed from the *tac* promoter from a multi-copy plasmid or when the level of SipA was probed from a strain in which this protein was tagged with the Flag epitope on the chromosome (Figures 1B,C). Further, direct probing of the endogenous protein with a SipA-specific antibody revealed that treatment with as low as 25 μM caused a clear reduction and 0.1 mM made the protein completely undetectable under our experimental conditions (Figure 1D).

Thymol-induced protein reduction was also observed for SipB under two experimental conditions in which the protein was expressed ectopically on a plasmid or from its cognate promoter on the chromosome (Figures 1E,F). We also examined the levels of HilA by measuring the activity of a HilA–LacZ fusion (Lin et al., 2008) and by detecting endogenous protein in wild-type *S*. Typhimurium. Treatment with 0.1 mM thymol caused severe reduction of both the HilA–LacZ fusion and the endogenous protein (Figure 1G). Taken together, these results demonstrate that thymol causes the reduction of a number of virulence proteins associated with the SPI-1.

**FIGURE 2:**
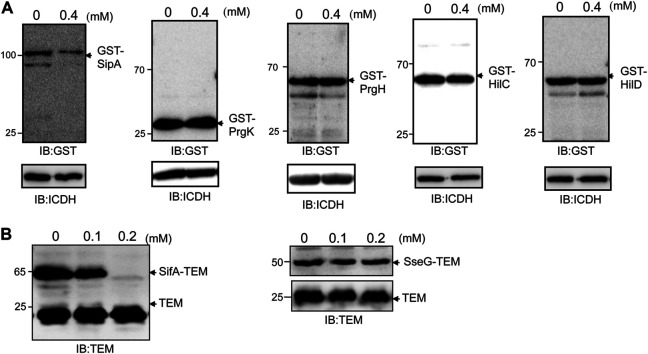
Thymol does not affect the stability of all proteins associated with SPI-1. **(A)** GST fusion of the indicted proteins was expressed in *S*. Typhimurium with or without thymol for 4 h, and the protein levels were evaluated by immunoblotting with a GST-specific antibody. ICDH was probed as a loading control. Note that only the protein level of GST-SipA decreased in cultures that received thymol. **(B)** The effects of thymol on the stability of SifA and SseG, two SPI-2 effectors. β-lactamase fused to SPI-2 effector SifA (left panel), or SseG (right panel) was expressed from a plasmid in wild-type *S*. Typhimurium in the presence of indicated concentrations of thymol for 4 h, and the protein was detected by immunoblotting with an antibody specific for β-lactamase. β-lactamase expressed from the plasmid backbone was used as a loading control. The experiments were repeated at least three times each with independent biological samples.

To determine the extent to which thymol affects the stability of *S*. Typhimurium virulence proteins, we further examined other important components of SPI-1. When expressed from an artificial promoter, thymol did not affect the stability of Prgk or PrgH, two proteins involved in the formation of the needle, nor did it affect the transcriptional factor HilC or HilD, which functions upstream of HilA (Ellermeier and Slauch, 2007) (Figure 2A). These results indicate that thymol does not indiscriminately reduce protein stability in *S*. Typhimurium.

The observation that thymol destabilizes a set of virulence proteins associated with SPI-1 prompted us to examine its effect on the activity of SPI-2, which codes for T3SS-2 that is required for intracellular replication, particularly in macrophages (Figueira and Holden, 2012). Because the expression of SPI-2 is induced by intracellular environment, which is more difficult to be mimicked in bacteriological media than SPI-1, we determined whether thymol affects the stability of two SPI-2 effectors, SifA and SseG (Figueira and Holden, 2012). *S*. Typhimurium strains expressing β-lactamase fusion to each of these proteins were constructed, and protein levels of the fusions were examined upon thymol treatment. The addition of 0.2 mM thymol led to the reduction of the SifA–lactamase but not the SseG–lactamase fusion (Figure 2B), indicating that thymol affects the stability of a subset of SPI-2 effectors.

### Thymol Induces Conformational Changes in SipA by Directly Interacting With Two Regions of This Effector

**FIGURE 3:**
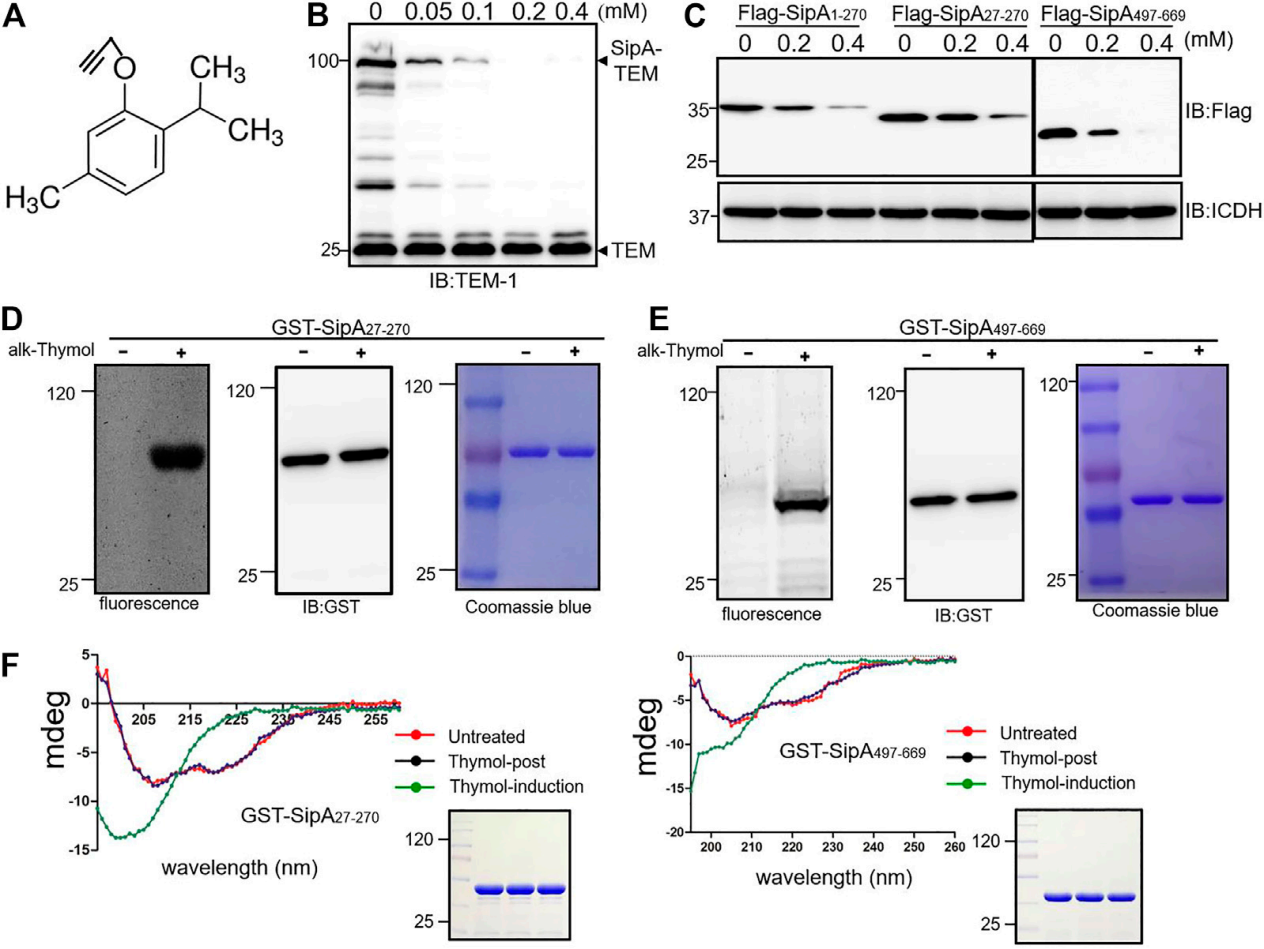
Thymol induces conformational changes in SipA by interacting with two independent regions. **(A)** The chemical structure of alkyne-thymol. **(B)** The alkyne modification did not affect the activity of thymol. Cells of the *S*. Typhimurium strain expressing the SipA–lactamase treated with different concentrations of alkyne-thymol were probed for the fusion protein by immunoblotting. Note that the reduction only occurs in the level of SipA–lactamase fusion but not in the lactamase expressed from the plasmid backbone. **(C)** Thymol affects the stability of the two soluble regions of SipA. Thymol was added to cultures of cells expressing the indicated SipA fragments fused to a Flag tag, and cell lysates resolved by SDS-PAGE were probed by immunoblotting with the Flag-specific antibody. ICDH was probed as a loading control. **(D,E)** The binding between alkyne-thymol and two fragments of SipA. GST fusion of SipA_27-270_ and SipA_497-669_ expressed in cells exposed to alkyne-thymol or solvent control reacted with azido-conjugated rhodamine was probed by fluorescence scanning. Note that in each case only protein from cells grown in the presence of alkyne-thymol retained the fluorescence signals. **(F)** Thymol induces conformational changes in SipA fragments. GST-SipA_27-270_ and GST-SipA_497-669_ purified from *E. coli* or its lysates exposed to thymol or solvent were analyzed by circular dichroism (CD) spectroscopy. Note that proteins from cells treated with thymol (green curve: thymol-induction) assume structures that differ greatly from those not exposed to thymol (red curve: untreated) or exposed to the compound (blue curve: thymol-post) after protein translation, untreated, thymol-post, and thymol-induction twisted antiparallel β-structures. The experiments were repeated at least three times each with independent biological samples.

To further probe the mechanism responsible for thymol-induced reduction of proteins associated with the SPI-1, we synthesized an alkyne-thymol derivative for click chemistry protein labeling experiments (Speers et al., 2003) (Figure 3A); this modification did not alter the effectiveness of thymol in the induction of SipA instability (Figure 3B). To simplify the study of the potential interactions between thymol and SipA, we first determined the responsiveness of regions of this effector to this compound. To this end, we expressed Flag-SipA_1-270_ and Flag-SipA_497-669,_ two soluble domains of this protein involved in chaperone-binding (Lilic et al., 2006) and actin nucleation (Galkin et al., 2002), respectively, in *S*. Typhimurium. Thymol was able to cause degradation of both fragments (Figure 3C). The removal of the first 27 residues that contain the signal necessary for T3SS-1-mediated secretion did not alter the responsiveness of SipA_1-270_ to thymol (Figure 3C). Thus, thymol interacts with at least two regions of SipA.

To detect the interactions between thymol and these two fragments of SipA, alkyne-thymol was added to *E. coli* cells expressing GST-tagged proteins. After expression induction by IPTG, GST-tagged SipA fragments were purified, and proteins potentially bound to alkyne-thymol were probed with azido-rhodamine, which reacts with the alkyne group. After the removal of unreacted molecules by chloroform/methanol extraction, samples resolved by SDS-PAGE were scanned for fluorescence signals or probed for the proteins. Fluorescence signals were detected only in samples receiving alkyne-thymol; no signal was detected in samples that did not receive the modified compound although the proteins were abundant as detected by immunoblotting or staining (Figures 3D,E). The observation that SDS and high temperature (100°C) did not disrupt the binding of thymol to these fragments of SipA suggests that thymol likely covalently interacts with this protein.

Next, we determined the consequence of thymol binding to the structure of SipA by circular dichroism (CD) spectroscopy analysis. GST-SipA_27-270_ and GST-SipA_497-669_ from untreated cells displayed characteristic structural features as previously reported (Mitra et al., 2000; Tsou et al., 2016) (Figure 3F). In contrast, proteins purified from cells treated with thymol exhibited significant changes in their CD spectra (Figure 3F). Importantly, when thymol was added to lysates of cells in which the expression of the protein had occurred, no change in the secondary structures was detected in either fragment, and in each case, the CD spectrum was identical to that of protein purified from cells that were never co-incubated with thymol (Figure 3F). These results suggest that the interactions between thymol and SipA that lead to protein destabilization only occur when the protein is being translated or is in its folding process.

### The Protease Lon Is Responsible for Thymol-Induced Virulence Protein Degradation

**FIGURE 4:**
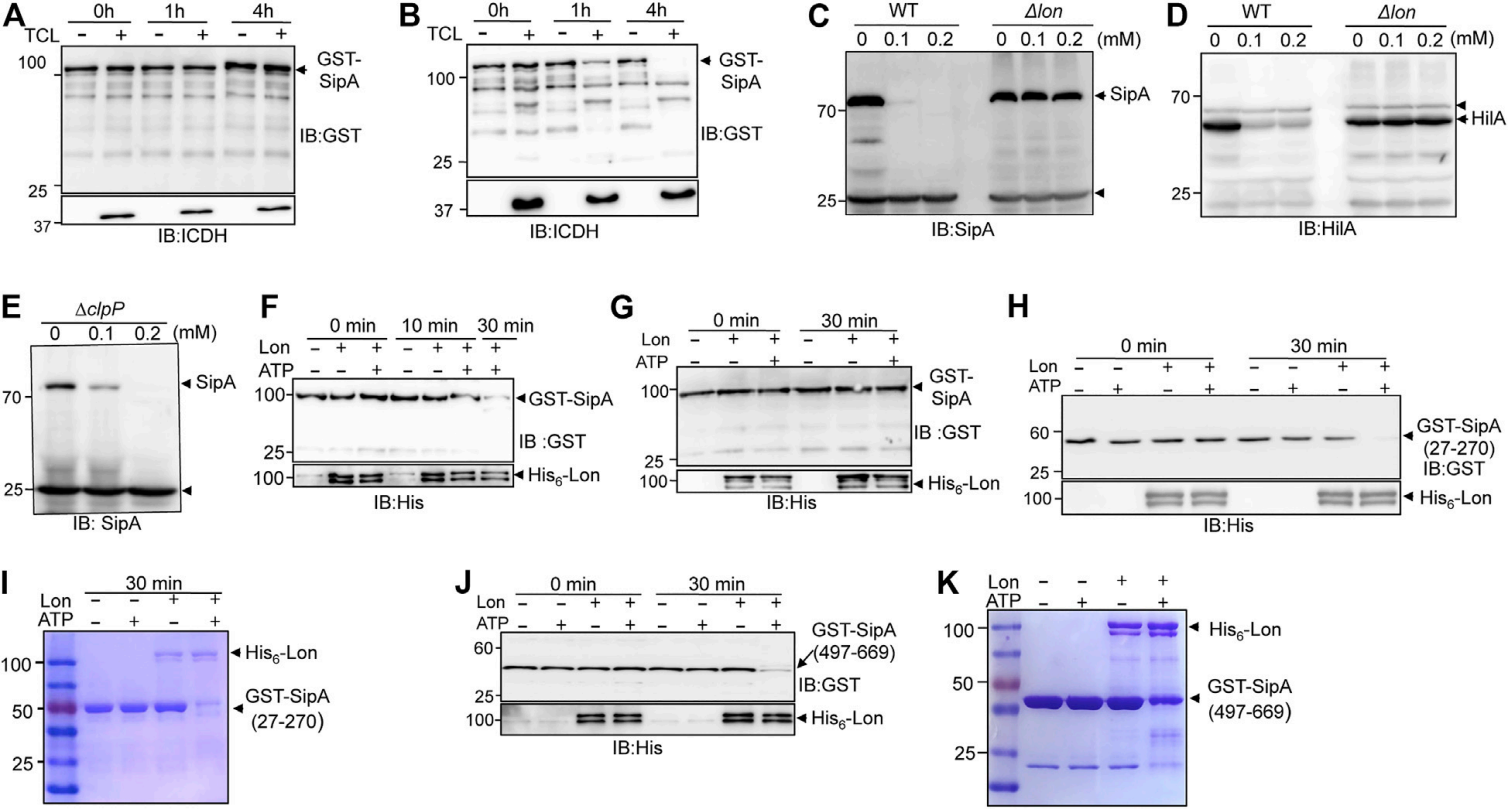
Lon protease degrades proteins targeted by thymol. **(A,B)** Degradation of SipA expressed in the presence of thymol by cell lysates of *S.* Typhimurium. GST-SipA purified from cultures containing thymol or its solvent was added to total cell lysates (TCL) of *S.* Typhimurium. Reactions terminated at the indicated time by SDS sample buffer were probed for the fusion protein by immunoblotting. ICDH in the reactions was probed to indicate the presence of equal amounts of TCL in relevant reactions. **(C–E)** The Lon but not the ClpP protease is required for thymol-induced degradation of SipA in *S.* Typhimurium. Total proteins of wild type, the *lon* **(C,D)**, or the *clpP* mutant **(E)** of *S.* Typhimurium grown in the presence of thymol or its solvent resolved by SDS-PAGE were probed for SipA or HilA by immunoblotting. Note that in the *clpP* mutant, thymol still caused robust reduction in SipA. The arrows indicate the bands nonspecifically recognized by the antibodies that serve as loading controls. **(F,G)** Lon only degrades GST-SipA from cells that have been exposed to thymol. GST-SipA purified from cultures supplemented with thymol **(F)** or its solvent **(G)** was mixed with His_6_-Lon, and ATP was added to a subset of samples to initiate the reactions. Reactions terminated at the indicated time points resolved by SDS-PAGE were probed for the fusion protein. 10% of the samples were probed for His_6_-Lon (lower panels). **(H–K)** The degradation of GST-SipA_27-270_ and GST-SipA_497-669_ by Lon. GST-SipA_27-270_ and GST-SipA_497-669_ purified from cultures grown in the presence of thymol were mixed with His6-Lon, and ATP was added to a subset of reactions to initiate the activity. Proteins of interest in reactions terminated at the indicated time points resolved by SDS-PAGE were probed by immunoblotting **(H,J)** or by Coomassie bright blue staining **(I,K)**. The experiments were repeated at least three times each with independent biological samples.

The observation that thymol treatment lowered the cellular level of SipA suggests that the binding leads to protein degradation. We tested such degradation in a cell-free system by adding GST-SipA purified from *E. coli* into total cell lysates of *S*. Typhimurium. Whereas the protein level of GST-SipA from untreated cells remained constant throughout the entire experimental duration (Figure 4A), the abundance of GST-SipA from thymol-treated cells became markedly lower after 1 h incubation and became undetectable when the incubation time was extended to 4 h (Figure 4B). Thus, SipA in complex with thymol is actively degraded by one or more protease systems. These results also indicate that the degradation machinery only recognizes SipA obtained from thymol-treated cells.

In bacteria, Lon and the ClpP are the two major protease systems responsible for energy-dependent degradation of most misfolded proteins (Gur and Sauer, 2008; Olivares et al., 2016). To determine whether any of them is involved in thymol-induced protein degradation, we obtained mutants defective in each of these protease systems and examined the level of SipA after thymol treatment. In the mutant lacking the Lon protease, even 0.2 mM thymol failed to induce SipA degradation (Figure 4C). This mutant is also defective in the thymol-induced degradation of HilA (Figure 4D). In contrast, SipA in the ∆*clpP* mutant was still robustly degraded in the presence of 0.1 mM thymol (Figure 4E), indicating that the Lon protease is responsible for degrading the proteins whose conformation has been altered by binding to thymol.

We further examined Lon-mediated degradation of SipA and its fragments in reactions containing purified proteins. In reactions containing recombinant Lon and a GST fusion of full-length SipA purified from cells treated with thymol, the degradation was evident after 10 min incubation, and the level of GST-SipA became almost undetectable when the reaction was allowed to proceed for 30 min (Figure 4F). Furthermore, in agreement with the fact that the activity of Lon requires energy (Gur and Sauer, 2008), no degradation occurred in reactions that did not receive ATP (Figure 4F). In contrast, Lon failed to degrade GST–SipA from cells that were never exposed to thymol even in reactions containing ATP (Figure 4G). Consistent with the results from experiments using live bacterial cells, both the SipA_27-270_ and SipA_497-669_ purified from thymol-treated cells were sensitive to Lon, again in an ATP-dependent manner (Figures 4H–K). These results demonstrate that after undergoing a conformational change, the target proteins of thymol are delivered to the Lon protease for degradation.

**FIGURE 5:**
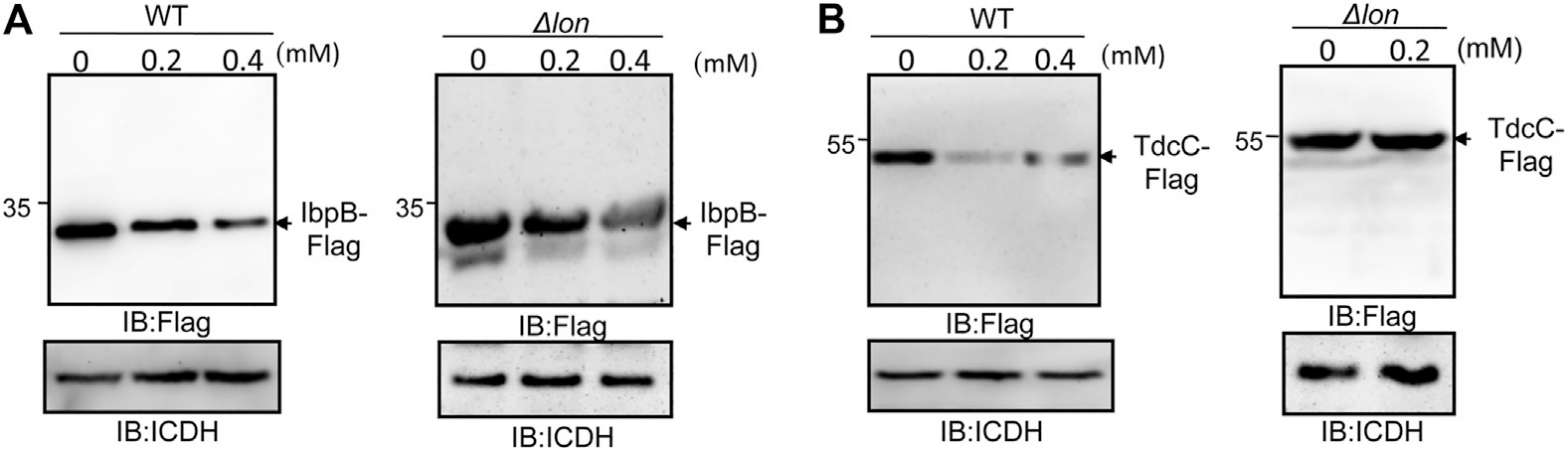
Thymol-induced degradation of two non-virulence proteins requires the Lon protease. Plasmids harboring Flag fused to the heat shock protein IbpB **(A)** and the threonine/serine transporter TdcC **(B)** were introduced into wild type *S*. Typhimurium and its *lon* mutant, respectively. Thymol was added to the cultures for 4 h prior to the detection of the fusion proteins by immunoblotting with a Flag-specific antibody. The bacterial ICDH was probed and served as a loading control. The experiments were repeated at least three times each with independent biological samples.

The abundance of at least 35 proteins not directly related to bacterial virulence was affected in cells treated with thymol (Tables 4, 5). We examined whether such reduction was caused by a mechanism that requires the Lon protease by testing the heat shock protein IbpB (Tomoyasu et al., 2003) and the threonine/serine transporter TdcC (Kroger et al., 2012). Plasmids that direct the production of IbpB-Flag and TdcC-Flag were introduced into wild-type and the *lon* mutant, respectively. Thymol was added to a subset of cultures for 4 h, and the proteins were detected by immunoblotting. Similar to SipA, thymol treatment led to a clear reduction in both fusion proteins in wild-type bacteria but not in the *lon* mutant (Figure 5), suggesting that thymol-induced destabilization of non-virulence proteins occurs by a mechanism similar to that for SipA.

**TABLE 5:** Protein with increased abundance in thymol-treated samples.

		Abundance^a^			
Gene	Protein description	Untreated	Thymol-treated	Fold change^b^	*p*-value^c^
torA	Trimethylamine-N-oxide reductase OS = *Salmonella* Typhimurium GN = torA PE = 3	2	124	72.3	0.000
torC	Trimethylamine N-oxide reductase OS = *Salmonella* Typhimurium GN = torC PE = 4	1.5	20	13.3	0.000
anE2	Putative N-acetylmannosamine-6-phosphate 2-epimerase 2 OS = *Salmonella* Typhimurium GN = nanE2 PE = 3 SV = 1	3.5	24	9.5	0.003
Rna	RNase I cleaves phosphodiester bond between any two nucleotidesOS = *Salmonella* Typhimurium GN = rna PE = 4 SV = 1	2	14	8.7	0.030
STM1540	Putative hydrolase OS = *Salmonella* Typhimurium GN = STM1540 PE = 4 SV = 1	4	28	7.7	0.004
fadA	3-ketoacyl-CoA thiolase OS = *Salmonella* Typhimurium GN = fadA PE = 3 SV = 1	2	12	6.7	0.002
gpmB	Probable phosphoglycerate mutase gpmB OS = *Salmonella* Typhimurium GN = gpmBPE = 3 SV = 1	7	28	6.3	0.030
fadD	Long-chain-fatty-acid-CoA ligase OS = *Salmonella* Typhimurium GN = fadD PE = 3	1.5	9	6.0	0.004
proW	Glycine betaine/L-proline transport system permease protein proW OS = *Salmonella* Typhimurium GN = proW PE = 3 SV = 2	2.5	14	6.0	0.001
A0SXM1	Beta-lactamase TEM-63 (Fragment) OS = *Salmonella* Typhimurium PE = 4 SV = 1	2	11	6.0	0.004
STM2006	Putative branched chain amino acid transport protein OS = *Salmonella* Typhimurium GN = STM2006 PE = 4 SV = 1	3.5	14	5.3	0.025
rhaD	Rhamnulose-1-phosphate aldolase OS = *Salmonella* Typhimurium GN = rhaD PE = 3	3.5	12	5.0	0.027
ybfF	Putative enzyme OS = *Salmonella* Typhimurium GN = ybfF PE = 4 SV = 1	3	15	5.0	0.024
STM1123	Putative periplasmic protein OS = *Salmonella* Typhimurium GN = STM1123 PE = 4	2	9	4.7	0.023
pitA	Low-affinity phosphate transporter OS = *Salmonella* Typhimurium GN = pitA PE = 4	1.5	7	4.7	0.023
yhbH	Probable sigma (54) modulation protein OS = *Salmonella* Typhimurium GN = yhbHPE = 3 SV = 1	3	10	4.7	0.023
yeiT	Uncharacterized oxidoreductase yeiT OS = *Salmonella* Typhimurium GN = yeiTPE = 4 SV = 1	11	33	4.2	0.017
Crl	Sigma factor-binding protein crl OS = *Salmonella* Typhimurium GN = crl PE = 2 SV = 1	3	12	4.0	0.020
pyrI	Aspartate carbamoyltransferase regulatory chain OS = *Salmonella* Typhimurium GN = pyrI PE = 3 SV = 3	1.5	4	2.7	0.020
T059	Putative adenine-specific DNA methylase OS = *Salmonella* Typhimurium GN = PSLT059 PE = 4 SV = 1	2	6	2.7	0.020
traD	Conjugative transfer: DNA transport OS = *Salmonella* Typhimurium GN = traD PE = 4SV = 1	1.5	4	2.7	0.020
treC	Trehalose-6-P hydrolase OS = *Salmonella* Typhimurium GN = treC PE = 4 SV = 1	4.5	10	2.7	0.020
trxC	Thioredoxin 2, redox factor OS = *Salmonella* Typhimurium GN = trxC PE = 4 SV = 1	2.5	7	2.7	0.008
yehS	Putative cytoplasmic protein OS = *Salmonella* Typhimurium GN = yehS PE = 4 SV = 1	1.5	4	2.7	0.020
yraL	Putative methyltransferase OS = *Salmonella* Typhimurium GN = yraL PE = 4 SV = 1	2.5	7	2.7	0.020
folE	GTP cyclohydrolase 1 OS = *Salmonella* Typhimurium GN = folE PE = 3 SV = 2	11	28	2.6	0.040
odC1	Superoxide dismutase [Cu-Zn] 1 OS = *Salmonella* Typhimurium GN = sodC1 PE = 1	9	23	2.6	0.017
hutH	Histidine ammonia-lyase OS = *Salmonella* Typhimurium GN = hutH PE = 3 SV = 1	21	51	2.5	0.017
spoT	(P)ppGpp synthetase II OS = *Salmonella* Typhimurium GN = spoT PE = 3 SV = 1	3.5	7	2.5	0.049
asmA	Suppressor of ompF assembly mutants OS = *Salmonella* Typhimurium GN = asmAPE = 4 SV = 1	6	16	2.4	0.014
STM3780	Fructose-bisphosphate aldolase OS = *Salmonella* Typhimurium GN = STM3780PE = 3 SV = 1	3	7	2.3	0.010
recR	Recombination protein recR OS = *Salmonella* Typhimurium GN = recR PE = 3 SV = 1	3	7	2.3	0.010
sufC	Putative transport protein OS = *Salmonella* Typhimurium GN = sufC PE = 3 SV = 1	3	7	2.3	0.010
ybhF	Putative ABC-type multidrug transport system, ATPase component OS = *Salmonella* Typhimurium GN = ybhF PE = 4 SV = 1	7	17	2.3	0.040
ybeB	Putative ACR, homolog of plant Iojap protein OS = *Salmonella* Typhimurium GN = ybeB PE = 4 SV = 1	18	35	2.3	0.047
mobA	Molybdopterin-guanine dinucleotide biosynthesis protein A OS = *Salmonella* Typhimurium GN = mobA PE = 3 SV = 1	5	10	2.2	0.035
nuoI	NADH-quinone oxidoreductase subunit I OS = *Salmonella* Typhimurium GN = nuoIPE = 3 SV = 1	13	28	2.2	0.030
lolD	Lipoprotein-releasing system ATP-binding protein lolD OS = *Salmonella* Typhimurium GN = lolD PE = 3 SV = 1	8	17	2.1	0.002
nagD	Putative phosphatase in N-acetylglucosamine metabolism OS = *Salmonella* Typhimurium GN = nagD PE = 4 SV = 1	13	26	2.1	0.027
pdxY	Pyridoxamine kinase OS = *Salmonella* Typhimurium GN = pdxY PE = 3 SV = 1	7	14	2.1	0.044
rpmH	50S ribosomal protein L34 OS = *Salmonella* Typhimurium GN = rpmH PE = 3 SV = 1	9	17	2.1	0.044

a Averaged spectral counts from three biological replicates.

b Fold change in protein abundance; positive or negative values indicate higher or lower levels in the thymol-treated samples comparing to untreated cells.

c *p*-values were calculated using paired Student’s *t*-test.

## Discussion

Although it has been reported that thymol could reduce the virulent gene expression in *S*. Typhimurium (Giovagnoni et al., 2020; Qi et al., 2020), the underlying mechanism is still unclear. Our results reveal that natural monoterpene phenol thymol targets multiple *S*. Typhimurium virulence factors for Lon protease degradation, thus protecting the host against the bacterial infection.

The IC_50_ of thymol in its inhibition of SipA-lactamase translocation by T3SS-1 of *S*. *typhimurium* is about 0.05 mM, which is at least 20 times lower than its lowest MIC (150 μg/ml or 1 mM) against *S*. Typhimurium (Chauhan and Kang, 2014; Marchese et al., 2016). Our results demonstrate that 11 out of 46 proteins affected by thymol are associated with SPI-1, which indicates its considerable specificity against *S*. Typhimurium virulence. Instead of inhibiting the activity of the T3SS-1 machinery itself, thymol targets proteins associated with the SPI-1 for degradation, including effectors, chaperones, and the regulatory protein HilA, an OmpR/ToxR family transcriptional regulator that controls the expression of a large number of virulence genes (Bajaj et al., 1995; Thijs et al., 2007). It is worth noting that the protein levels of some known targets of HilA did not significantly decrease in thymol-treated cells in our proteomic analysis. Such discrepancies may arise from the reproducibility of the proteomic approaches or that the expression of these proteins is under the control of multiple layers of regulation, which is a common phenomenon in *S*. Typhimurium (Ellermeier and Slauch, 2007; Smith et al., 2016). Nevertheless, because mutants lacking one or more effectors such as SipA and SipB are partially defective in virulence (Hersh et al., 1999; Zhou et al., 1999), the protection seen in cell invasion clearly attributes to the additive effects of lowering the level of both effectors and the regulatory protein, which governs the expression of multiple effectors and other proteins involved in virulence (Bajaj et al., 1995; Ellermeier and Slauch, 2007; Thijs et al., 2007).

The fact that the putative complexes formed by thymol and fragments of SipA endured SDS and β-mercaptoethanol at high temperature implied that the compound is covalently linked to its target molecules. Alternatively, one or more of its derivatives generated in bacterial cells may directly bind these proteins by covalent bonds. The two regions in SipA that independently interact with thymol do not share detectable homology, nor do the proteins that appeared to be targeted by this compound. The mechanism underlying the recognition of multiple proteins without similarity warrants further studies. It is possible that thymol is inserted into a transitional configuration shared by all of its targets during protein folding, thus preventing them from assuming the proper final structures. This notion is consistent with the fact that thymol did not induce conformational changes in SipA if it was added after translation of the proteins had been complete (Figure 4F). The stable interactions between thymol and its target proteins may allow future work to determine the structures of the protein–drug complexes, which should provide not only the molecular details for the binding but also the information useful in the modification of this compound to increase its efficacy and specificity. Furthermore, the finding that thymol directs virulence factors for protease degradation opens a research avenue to develop anti-virulence strategies.

## Data Availability

BALB/c mice were obtained from the Experimental Animal Center of Jilin University. All procedures involving animals were carried out in accordance with the Guidelines for the Use of Animals in Research, issued by Jilin University. The animal experiment protocols were ethically approved by the IACUC of Jilin University.

## References

[B1] Agbor T. A. McCormick B. A. (2011). Salmonella Effectors: Important Players Modulating Host Cell Function during Infection. Cell Microbiol 13 (12), 1858–1869. 10.1111/j.1462-5822.2011.01701.x 21902796 PMC3381885

[B2] Bajaj V. Hwang C. Lee C. A. (1995). hilA Is a Novel ompR/toxR Family Member that Activates the Expression of *Salmonella typhimurium* Invasion Genes. Mol. Microbiol. 18 (4), 715–727. 10.1111/j.1365-2958.1995.mmi_18040715.x 8817493

[B3] Boddicker J. D. Jones B. D. (2004). Lon Protease Activity Causes Down-Regulation of Salmonella Pathogenicity Island 1 Invasion Gene Expression after Infection of Epithelial Cells. Infect. Immun. 72 (4), 2002–2013. 10.1128/iai.72.4.2002-2013.2004 15039320 PMC375200

[B4] Cabarkapa I. Colovic R. Duragic O. Popovic S. Kokic B. Milanov D. (2019). Anti-biofilm Activities of Essential Oils Rich in Carvacrol and Thymol against Salmonella Enteritidis. Biofouling 35 (3), 361–375. 10.1080/08927014.2019.1610169 31088182

[B5] Chauhan A. K. Kang S. C. (2014). Thymol Disrupts the Membrane Integrity of Salmonella Ser. Typhimurium *In Vitro* and Recovers Infected Macrophages from Oxidative Stress in an *Ex Vivo* Model. Res. Microbiol. 165 (7), 559–565. 10.1016/j.resmic.2014.07.001 25049168

[B6] Cherepanov P. P. Wackernagel W. (1995). Gene Disruption in Escherichia coli: TcR and KmR Cassettes with the Option of Flp-Catalyzed Excision of the Antibiotic-Resistance Determinant. Gene 158 (1), 9–14. 10.1016/0378-1119(95)00193-a 7789817

[B7] Costa T. R. D. Felisberto-Rodrigues C. Meir A. Prevost M. S. Redzej A. Trokter M. (2015). Secretion Systems in Gram-Negative Bacteria: Structural and Mechanistic Insights. Nat. Rev. Microbiol. 13 (6), 343–359. 10.1038/nrmicro3456 25978706

[B8] Datsenko K. A. Wanner B. L. (2000). One-step Inactivation of Chromosomal Genes in Escherichia coli K-12 Using PCR Products. Proc. Natl. Acad. Sci. 97 (12), 6640–6645. 10.1073/pnas.120163297 10829079 PMC18686

[B9] Ellermeier J. R. Slauch J. M. (2007). Adaptation to the Host Environment: Regulation of the SPI1 Type III Secretion System in Salmonella enterica Serovar Typhimurium. Curr. Opin. Microbiol. 10 (1), 24–29. 10.1016/j.mib.2006.12.002 17208038

[B10] Figueira R. Holden D. W. (2012). Functions of the Salmonella Pathogenicity Island 2 (SPI-2) Type III Secretion System Effectors. Microbiology 158 (Pt 5), 1147–1161. 10.1099/mic.0.058115-0 22422755

[B11] Galkin V. E. Orlova A. VanLoock M. S. Zhou D. Galán J. E. Egelman E. H. (2002). The Bacterial Protein SipA Polymerizes G-Actin and Mimics Muscle Nebulin. Nat. Struct. Biol. 9 (7), 518–521. 10.1038/nsb811 12055622

[B12] Giovagnoni G. Rossi B. Tugnoli B. Ghiselli F. Bonetti A. Piva A. (2020). Thymol and Carvacrol Downregulate the Expression of *Salmonella typhimurium* Virulence Genes during an *In Vitro* Infection on Caco-2 Cells. Microorganisms 8 (6), 862. 10.3390/microorganisms8060862 PMC7355688 32517327

[B13] Gulig P. A. Curtiss R. 3rd (1987). Plasmid-associated Virulence of *Salmonella typhimurium* . Infect. Immun. 55 (12), 2891–2901. 10.1128/iai.55.12.2891-2901.1987 3316027 PMC260003

[B14] Gur E. Sauer R. T. (2008). Recognition of Misfolded Proteins by Lon, a AAA+ Protease. Genes Develop. 22 (16), 2267–2277. 10.1101/gad.1670908 18708584 PMC2518814

[B15] Hersh D. Monack D. M. Smith M. R. Ghori N. Falkow S. Zychlinsky A. (1999). The Salmonella Invasin SipB Induces Macrophage Apoptosis by Binding to Caspase-1. Proc. Natl. Acad. Sci. 96 (5), 2396–2401. 10.1073/pnas.96.5.2396 10051653 PMC26795

[B16] Hu M. Liu Y. Yu K. Liu X. (2014). Decreasing the Amount of Trypsin in In-Gel Digestion Leads to Diminished Chemical Noise and Improved Protein Identifications. J. Proteomics 109, 16–25. 10.1016/j.jprot.2014.06.017 24984109

[B17] Johnson R. Mylona E. Frankel G. (2018). Typhoidal Salmonella: Distinctive Virulence Factors and Pathogenesis. Cel Microbiol 20 (9), e12939. 10.1111/cmi.12939 30030897

[B18] Kroger C. Dillon S. C. Cameron A. D. S. Papenfort K. Sivasankaran S. K. Hokamp K. (2012). The Transcriptional Landscape and Small RNAs of Salmonella enterica Serovar Typhimurium. Proc. Natl. Acad. Sci. 109 (20), E1277–E1286. 10.1073/pnas.1201061109 22538806 PMC3356629

[B19] Lau H.-T. Suh H. W. Golkowski M. Ong S.-E. (2014). Comparing SILAC- and Stable Isotope Dimethyl-Labeling Approaches for Quantitative Proteomics. J. Proteome Res. 13 (9), 4164–4174. 10.1021/pr500630a 25077673 PMC4156256

[B20] Lilic M. Vujanac M. Stebbins C. E. (2006). A Common Structural Motif in the Binding of Virulence Factors to Bacterial Secretion Chaperones. Mol. Cel. 21 (5), 653–664. 10.1016/j.molcel.2006.01.026 16507363

[B21] Lin D. Rao C. V. Slauch J. M. (2008). The Salmonella SPI1 Type Three Secretion System Responds to Periplasmic Disulfide Bond Status via the Flagellar Apparatus and the RcsCDB System. J. Bacteriol. 190 (1), 87–97. 10.1128/jb.01323-07 17951383 PMC2223759

[B22] Liu H. Sadygov R. G. Yates J. R. 3rd (2004). A Model for Random Sampling and Estimation of Relative Protein Abundance in Shotgun Proteomics. Anal. Chem. 76 (14), 4193–4201. 10.1021/ac0498563 15253663

[B23] Luo Z.-Q. Farrand S. K. (1999). Signal-dependent DNA Binding and Functional Domains of the Quorum-sensing Activator TraR as Identified by Repressor Activity. Proc. Natl. Acad. Sci. 96 (16), 9009–9014. 10.1073/pnas.96.16.9009 10430886 PMC17723

[B24] Luo Z.-Q. Isberg R. R. (2004). Multiple Substrates of the Legionella pneumophila Dot/Icm System Identified by Interbacterial Protein Transfer. Proc. Natl. Acad. Sci. 101 (3), 841–846. 10.1073/pnas.0304916101 14715899 PMC321768

[B25] Lv Q. Li S. Wei H. Wen Z. Wang Y. Tang T. (2020). Identification of the Natural Product Paeonol Derived from Peony Bark as an Inhibitor of the Salmonella enterica Serovar Typhimurium Type III Secretion System. Appl. Microbiol. Biotechnol. 104 (4), 1673–1682. 10.1007/s00253-019-10290-7 31897522

[B26] Marchese A. Orhan I. E. Daglia M. Barbieri R. Di Lorenzo A. Nabavi S. F. (2016). Antibacterial and Antifungal Activities of Thymol: A Brief Review of the Literature. Food Chem. 210, 402–414. 10.1016/j.foodchem.2016.04.111 27211664

[B27] Mitra K. Zhou D. Galan J. E. (2000). Biophysical Characterization of SipA, an Actin-Binding Protein from Salmonella enterica. FEBS Lett. 482 (1-2), 81–84. 10.1016/s0014-5793(00)02040-8 11018527

[B28] Olivares A. O. Baker T. A. Sauer R. T. (2016). Mechanistic Insights into Bacterial AAA+ Proteases and Protein-Remodelling Machines. Nat. Rev. Microbiol. 14 (1), 33–44. 10.1038/nrmicro.2015.4 26639779 PMC5458636

[B29] Pendergrass H. A. May A. E. (2019). Natural Product Type III Secretion System Inhibitors. Antibiotics (Basel) 8 (4). 10.3390/antibiotics8040162 PMC6963908 31554164

[B30] Qi Y. Zhao W. Wang T. Pei F. Yue M. Li F. (2020). Proteomic Analysis of the Antimicrobial Effects of Sublethal Concentrations of Thymol on Salmonella enterica Serovar Typhimurium. Appl. Microbiol. Biotechnol. 104 (8), 3493–3505. 10.1007/s00253-020-10390-9 32072194

[B31] Rasko D. A. Sperandio V. (2010). Anti-virulence Strategies to Combat Bacteria-Mediated Disease. Nat. Rev. Drug Discov. 9 (2), 117–128. 10.1038/nrd3013 20081869

[B32] Sang Y. Ren J. Ni J. Tao J. Lu J. Yao Y.-F. (2016). Protein Acetylation Is Involved inSalmonella entericaSerovar Typhimurium Virulence. J. Infect. Dis. 213 (11), 1836–1845. 10.1093/infdis/jiw028 26810370

[B33] Smith C. Stringer A. M. Mao C. Palumbo M. J. Wade J. T. (2016). Mapping the Regulatory Network for Salmonella enterica Serovar Typhimurium Invasion. mBio 7 (5). 10.1128/mbio.01024-16 PMC5013294 27601571

[B34] Speers A. E. Adam G. C. Cravatt B. F. (2003). Activity-based Protein Profiling *In Vivo* Using a Copper(i)-Catalyzed Azide-Alkyne [3 + 2] Cycloaddition. J. Am. Chem. Soc. 125 (16), 4686–4687. 10.1021/ja034490h 12696868

[B35] Sundin C. Zetterstrom C. E. Vo D. D. Brkljaca R. Urban S. Elofsson M. (2020). Exploring Resveratrol Dimers as Virulence Blocking Agents - Attenuation of Type III Secretion in Yersinia Pseudotuberculosis and Pseudomonas aeruginosa. Sci. Rep. 10 (1), 2103. 10.1038/s41598-020-58872-0 32034212 PMC7005745

[B36] Thijs I. M. V. De Keersmaecker S. C. J. Fadda A. Engelen K. Zhao H. McClelland M. (2007). Delineation of the Salmonella enterica Serovar Typhimurium HilA Regulon through Genome-wide Location and Transcript Analysis. J. Bacteriol. 189 (13), 4587–4596. 10.1128/jb.00178-07 17483226 PMC1913449

[B37] Tomoyasu T. Takaya A. Sasaki T. Nagase T. Kikuno R. Morioka M. (2003). A New Heat Shock Gene, agsA , Which Encodes a Small Chaperone Involved in Suppressing Protein Aggregation in Salmonella enterica Serovar Typhimurium. J. Bacteriol. 185 (21), 6331–6339. 10.1128/jb.185.21.6331-6339.2003 14563868 PMC219406

[B38] Tsou L. K. Lara-Tejero M. RoseFigura J. Zhang Z. J. Wang Y.-C. Yount J. S. (2016). Antibacterial Flavonoids from Medicinal Plants Covalently Inactivate Type III Protein Secretion Substrates. J. Am. Chem. Soc. 138 (7), 2209–2218. 10.1021/jacs.5b11575 26847396 PMC4831573

[B39] Xu L. Shen X. Bryan A. Banga S. Swanson M. S. Luo Z. Q. (2010). Inhibition of Host Vacuolar H+-ATPase Activity by a Legionella pneumophila Effector. PLoS Pathog. 6 (3), e1000822. 10.1371/journal.ppat.1000822 20333253 PMC2841630

[B40] Zhang Y. Liu Y. Qiu J. Luo Z. Q. Deng X. (2018). The Herbal Compound Thymol Protects Mice from Lethal Infection by Salmonella Typhimurium. Front. Microbiol. 9, 1022. 10.3389/fmicb.2018.01022 29867906 PMC5968388

[B41] Zhou D. Mooseker M. S. Galan J. E. (1999). Role of the S. typhimurium Actin-Binding Protein SipA in Bacterial Internalization. Science 283 (5410), 2092–2095. 10.1126/science.283.5410.2092 10092234

